# Astrocyte reactivity to Fas activation is attenuated in TIMP-1 deficient mice, an *in vitro *study

**DOI:** 10.1186/1471-2202-6-68

**Published:** 2005-11-29

**Authors:** Crystel Ogier, Rita Creidy, José Boucraut, Paul D Soloway, Michel Khrestchatisky, Santiago Rivera

**Affiliations:** 1 Neurobiologie des Interactions Cellulaires et Neurophysiopathologie (NICN), CNRS UMR 6184. Université de la Méditerranée. Faculté de Médecine de Marseille, IFR Jean Roche. Bd. Pierre Dramard, 13916, Marseille cedex 20, France; 2 Division of Nutritional Sciences College of Agriculture and Life Sciences, Cornell University 108 Savage Hall Ithaca, NY 14853-6301 USA

## Abstract

**Background:**

Tissue inhibitor of metalloproteinases-1 (TIMP-1) is a multifunctional secreted protein with pleiotropic actions, including the inhibition of matrix metalloproteinases (MMPs), cell death/survival and growth promoting activities. After inflammatory challenge, the levels of TIMP-1 are highly and selectively upregulated in astrocytes among glial cells, but little is know about its role in these neural cells. We investigated the influence of TIMP-1 null mutation in the reactivity of cultured astrocytes to pro-inflammatory stimuli with TNF-α and anti-Fas antibody.

**Results:**

When compared to WT, mutant astrocytes displayed an overall increased constitutive gelatinase expression and were less responsive to Fas-mediated upregulation of MMP-9, of monocyte chemoattractant protein-1 (MCP-1) and of intercellular cell adhesion molecule-1 (ICAM-1), all markers of astrocyte inflammatory response. In contrast, TNF-α treatment induced all these factors similarly regardless of the astrocyte genotype. The incorporation of ^3^H-thymidin, a marker of cell proliferation, increased in wild-type (WT) astrocytes after treatment with anti-Fas antibody or recombinant TIMP-1 but not in mutant astrocytes. Finally, lymphocyte chemotaxis was differentially regulated by TNF-α in WT and TIMP-1 deficient astrocytes.

**Conclusion:**

We provide evidence that the alteration of the MMP/TIMP balance in astrocytes influences their reactivity to pro-inflammatory stimuli and that Fas activation modulates the expression of members of the MMP/TIMP axis. We hypothesise that the Fas/FasL transduction pathway and the MMP/TIMP system interact in astrocytes to modulate their inflammatory response to environmental stimuli.

## Background

Injury in the central nervous system (CNS) is generally accompanied by an inflammatory reaction that involves mainly microglia and astrocytes. The latter are the most abundant cells in the CNS and their contribution to the pathological outcome is still a matter of debate. In response to injury, cytokines and chemokines trigger astrocyte proliferation and migration into the lesioned area where astrocytes contribute to the formation of the glial scar that inhibits axonal regeneration in the CNS [1]. Characteristic features of reactive astrocytes are morphological changes with cell body hypertrophy and increased expression of a number of proteins absent or weakly expressed in their resting state. Among these proteins, the MMPs and the TIMPs are highly upregulated in reactive astrocytes. TIMP-1 is a 31 kDa multifunctional secreted glycoprotein that possesses, in addition to its MMP inhibitor activity, growth promoting activities in a number of non neural cells [2-4]. We first demonstrated that TIMP-1 is massively and sequentially upregulated in cortical areas of rat brain after kainate-induced seizures, first in resistant neurons and subsequently in reactive astrocytes [5]. Selective TIMP-1 upregulation in astrocytes has also been reported after experimental autoimmune encephalomyelitis [6] or cerebral ischemia [7]. Interestingly, none of the aforementioned studies reported TIMP-1 expression in reactive microglial cells, highlighting the possibility of a specific role for TIMP-1 in astrocytes among glial cells. In cultured astrocytes, TIMP-1 is induced in response to a number of pro-inflammatory stimuli, including cytokines activated in the injured brain such as TNF-α or IL-1β˙
 MathType@MTEF@5@5@+=feaafiart1ev1aaatCvAUfKttLearuWrP9MDH5MBPbIqV92AaeXatLxBI9gBaebbnrfifHhDYfgasaacH8akY=wiFfYdH8Gipec8Eeeu0xXdbba9frFj0=OqFfea0dXdd9vqai=hGuQ8kuc9pgc9s8qqaq=dirpe0xb9q8qiLsFr0=vr0=vr0dc8meaabaqaciGacaGaaeqabaqabeGadaaakeaaiiaacuWFYoGygaqgaaaa@2E74@ [8-10], LPS [11] or after transient exposure to activated T lymphocytes [12]. Nevertheless, the effects of TIMP-1 in astrocytes are still largely unknown. We thus investigated the influence of TIMP-1 null mutation [13] on the response of cultured astrocytes to two cytokines of the TNF superfamily know to be induced in similar physiopathological conditions than TIMP-1. Notably, TNF-α and the Fas/FasL system are known to be death molecules for different cell types [14-16], but act as pro-inflammatory agents in astrocytes [17-20] the latter being resistant to Fas mediated cytotoxic effects [21-23]. In addition, it is known MMPs may regulate the activity of the TNF and Fas systems by proteolytic cleavage of some of their members, including TNF-α [24], TNF-R [25], Fas-L [26,27] and Fas [28].

We provide evidence that the absence of TIMP-1 prevents the induction of MMP-9 and of inflammatory markers such as ICAM-1 or MCP-1 after Fas activation and that the mutant astrocytes proliferate less than the wild type in response to cytokines and to TIMP-1. TIMP-1 null mutation is also accompanied by an increased constitutive expression of gelatinases, mainly MMP-2. Altogether, these data indicate that the absence of TIMP-1 specifically attenuates the inflammatory response of astrocytes triggered by Fas but not by TNF-α and suggest that the MMP/TIMP balance is an important determinant in the pro-inflammatory effects of some members of the TNF family.

## Results

### Characterisation of astrocyte cultures

**Figure 1:**
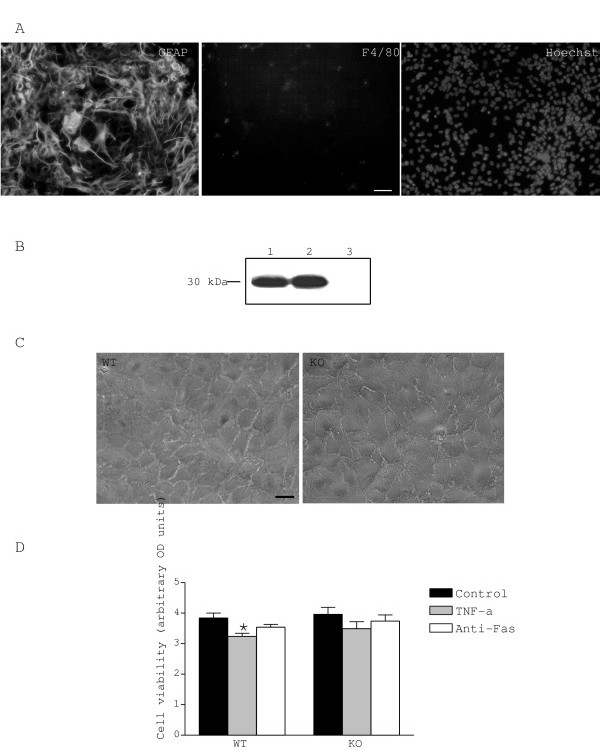
Characterisation of astrocyte cultures. **A. **Epifluorescence photomicrographs showing combined labelling with anti-GFAP and anti-F4/80 antibodies, and nuclear intercalant Hoechst. Astrocyte culture were charaterised as being greater than 95% pure by counting GFAP-positive cells and F4/80-positive cells (microglia) over the total number of cells stained by Hoechst. **B. **Expression of TIMP-1 in astrocytes was assessed by western blot; lane 1: mouse recombinant TIMP-1, lane 2: supernatant of WT astrocytes and lane 3: supernatant of KO astrocytes. **C. **Phase contrast photomicrographs of astrocyte cultures. No apparent morphological differences were seen between WT and KO astrocytes. **D. **Effect of treatments on cell viability. Only TNF-α treatment induced a mild cytotoxic effect on WT astrocytes. Values represent the mean +/- s.e.m. of at least three independent experiments. Statistical differences were determined by Student's *t*-test when comparing treatments to normalised values (* p < 0.05). Scale bar represents 50 μm.

Astrocyte cultures were characterised as being greater than 95% pure by counting GFAP positive cells over the total number of cells stained by Hoechts # 33258. Microglial cells stained with F4/80 constituted the majority of GFAP negative cells (Fig. 1A). We confirmed by western blot that WT astrocytes constitutively expressed TIMP-1 and that the protein was absent in astrocytes from KO mice (Fig. 1B). As shown in Fig. 1C, no morphological differences were observed between astrocytes from WT or KO mice and the density of confluent cells was equivalent. This observation was supported by the finding that the MTT levels were identical in untreated WT and TIMP-1 KO cultures. Parallel experiments using propidium iodide as a cell death marker showed no differences in the constitutive cell death of untreated astrocytes (2–3%) between WT and mutant mice (results not shown). We found a mild reduction (16%) in mitochondrial activity by the test MTT only in WT astrocytes treated by TNF-α and no effect after anti-Fas antibody exposure (Fig. 1D). We confirmed previous data [21-23] demonstrating the absence of anti-Fas toxicity in astrocytes.

### TIMP-1 null mutation causes altered expression of MMP-2 and MMP-9

**Figure 2:**
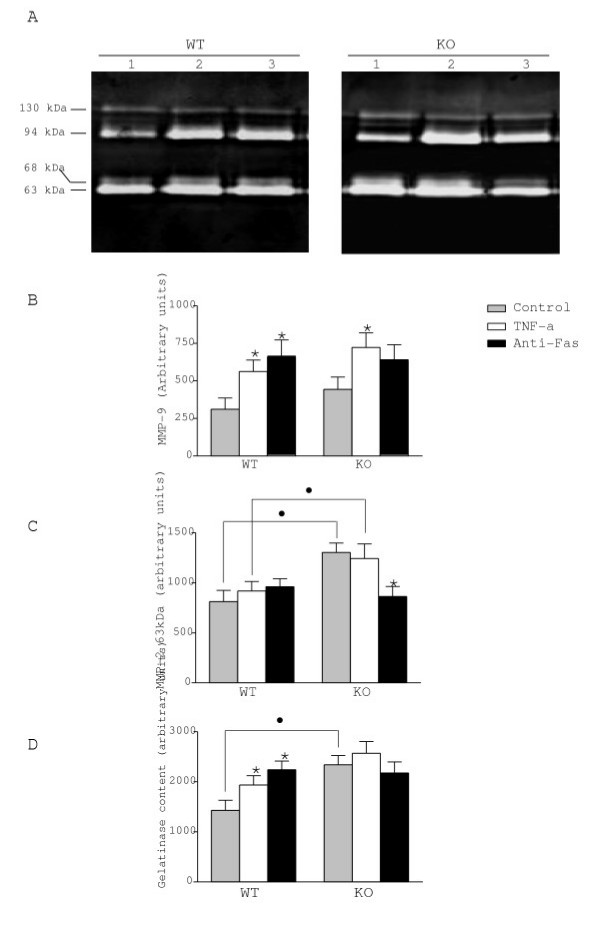
Expression of MMP-9 and MMP-2 in astrocytes. **A. **Gelatine zymograms from culture supernatants of WT and KO astrocytes in control condition (lane 1) or after treatment with TNF-α (lane 2) and anti-Fas antibody (lane 3). Quantitative expression of the 94 kDa (MMP-9) (**B**) and the 63 kDa form of MMP-2 (**C**). Constitutive levels of MMP-2 in KO were higher than in WT astrocytes. MMP-9 was up-regulated by TNF-α in both strains of astrocytes. Fas activation significantly induced MMP-9 expression in WT astrocytes but not in KO astrocytes. **D. **Total gelatinase expression detected in gel zymography. The total gelatinase expression was higher in KO astrocytes as compared to WT astrocytes. TNF-α and anti-Fas treatments increased total gelatinase expression only in WT astrocytes. Values represent the mean +/- s.e.m. of at least three independent experiments. Statistical differences were determined by Student's *t*-test when comparing treatments to control cells within a given genotype (* p < 0.05) or KO vs WT cells (● p < 0.05).

It is widely accepted that astrocytes express soluble MMP-2 and 9 (also known as gelatinases A and B, respectively) and TIMP-1. As TIMP-1 is the preferential inhibitor of MMP-9 and a good inhibitor of MMP-2, we used gel zymography to evaluate whether TIMP-1 depletion would influence the expression of both proteinases in the supernatants of WT and KO cultures after treatments. In control cultures, gel zymography (Fig. 2A) revealed the expected MMP-9 94 kDa band, and a predominant band at 63 kDa and a weaker band at 68 kDa, both of them coherent with expected molecular weights for MMP-2 active forms. We also detected a conspicuous band at 130 kDa. The constitutive levels of MMP-2 were more than twice the levels of MMP-9 in both WT and mutant astrocytes. TIMP-1 null mutation had no significant effect on constitutive MMP-9 expression, but increased the baseline levels of MMP-2 by 60%, as compared with WT astrocytes (Fig. 2C). In the latter, TNF-α and Fas activation significantly increased MMP-9 expression (80% and 110%, respectively). In the KO astrocytes, MMP-9 levels were upregulated only after exposure to TNF-α, while Fas activation had no significant effect (Fig. 2B). Treatments did not change the levels of MMP-2 except the anti-Fas antibody which reduced MMP-2 levels in KO astrocytes. When considering the total pool of gelatinases detected by gel zymography, we found that its constitutive level was higher in the KO astrocytes and increased with TNF-α and anti-Fas antibody in the WT astrocytes (Fig. 2D). Phenanthroline and BB-3103 abolished gelatinase activity, indicating that it was generated by metalloproteinases (not shown).

### ICAM-1 and MCP-1 are differentially induced in WT and TIMP-1 KO astrocytes

**Figure 3:**
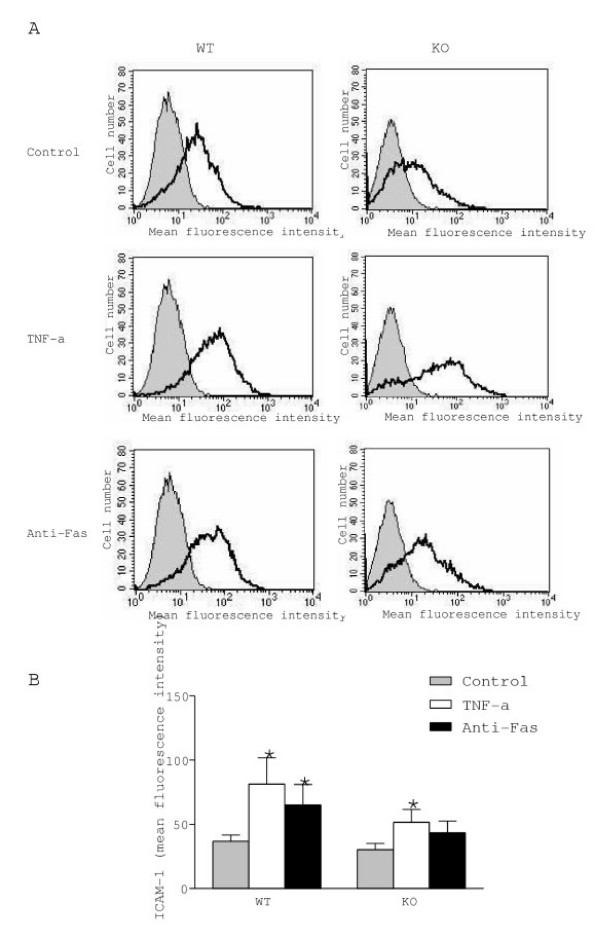
Expression of ICAM-1 in WT and KO astrocytes. **A. **Plots of flow cytometry after treatment with TNF-α or anti-Fas antibody. **B. **Quantification of ICAM-1 expression. ICAM-1 levels were up-regulated by TNF-α in astrocytes from WT and KO mice, whereas Fas activation increased ICAM-1 expression only in WT astrocytes. Values represent the mean +/- s.e.m. of at least three independent experiments. Statistical differences were determined by Student's *t*-test when comparing treatments to control cells (* p < 0.05).

**Figure 4:**
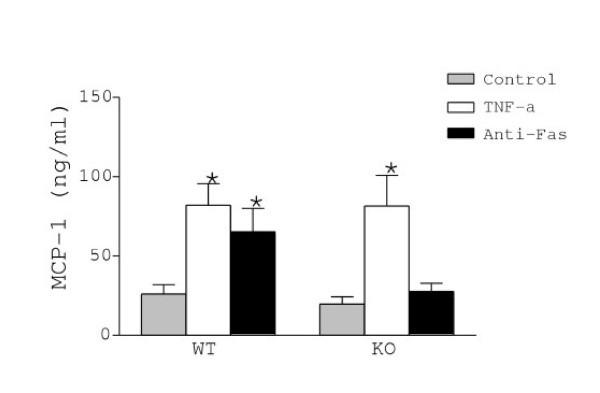
Release of MCP-1 by WT and KO astrocytes was measured in supernatants by ELISA. MCP-1 release was up-regulated by TNF-α in astrocytes from WT and KO mice, whereas Fas activation increased MCP-1 release only in WT astrocytes. Values represent the mean +/- s.e.m. of at least three independent experiments. Statistical differences were determined by Student's *t*-test when comparing treatments to control cells (* p < 0.05).

Astrocytes are the most abundant cells of the brain and express cytokines and adhesion molecules involved in different phases of neuroinflammation, including leukocyte recruitment in the CNS. Among these proteins, ICAM-1 and MCP-1 are strongly up-regulated during neuroinflammation, and preferentially in astrocytes [29-32]. Accordingly, both proteins are considered as markers of inflammatory response. In addition, the activities of ICAM-1 and MCP-1 are modulated by metalloproteinases [33,34]. For these reasons we examined whether the imbalance in the MMP/TIMP ratio observed in the mutant cultures would affect the levels of ICAM-1 and MCP-1 in astrocytes. As illustrated in figures 3 and 4, we found no differences in the constitutive expression of membrane bound ICAM-1 or release of MCP-1 between WT and KO cultures. However, exposure to TNF-α increased ICAM-1 levels in both WT and KO cultures (120% and 80%, respectively) (Fig. 3). An even higher upregulation of MCP-1 levels was observed after TNF-α in WT (215%) and KO (310%) (Fig. 4). In contrast, Fas activation upregulated ICAM-1 (70%) and MCP-1 (150%) levels only in WT mice, but not in TIMP-1 deficient astrocytes.

### KO astrocytes proliferate less than WT astrocytes in response to TNF-α and anti-Fas

**Figure 5:**
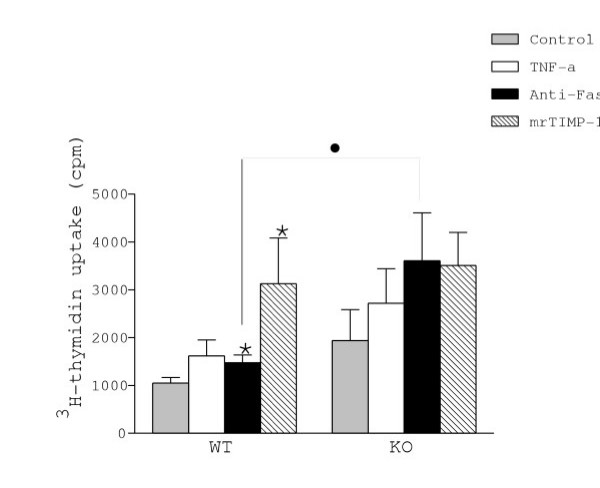
Astrocyte proliferation was estimated by measuring ^3^H-thymidin uptake, expressed in cpm. Note that constitutive uptake was higher in KO than in WT astrocytes. Both anti-Fas antibody and mrTIMP-1 increased proliferation of WT astrocytes but not of KO astrocytes. Values represent the mean +/- s.e.m. of at least three independent experiments. Statistical differences were determined by Student's t test when comparing treatments to control cells with the same genotype (* p < 0.05) or KO vs WT cells (● p < 0.05).

Since cell proliferation is a hallmark of glial response to neuroinflammatory challenge, we measured the incorporation of ^3^H-thymidin, as an index of proliferation, into first passage synchronised astrocytes in response to cytokines and mrTIMP-1 (Fig. 5). We show that mrTIMP-1 induced the highest increase (200%) in proliferation of WT cells. Even though the levels of ^3^H-thymidin uptake in KO astrocytes treated with TIMP-1 were equivalent to those of WT astrocytes, statistical significance with respect to the untreated KO control was not reached, possibly because of a high level of constitutive ^3^H-thymidin incorporation in the mutant astrocytes. TNF-α did not significantly stimulate ^3^H-thymidin uptake in WT and KO astrocytes, unlike anti-Fas antibody that induced a significant 40% increase in WT but not in KO astrocytes.

### Effect of WT and KO astrocytes on lymphocyte recruitment

**Figure 6:**
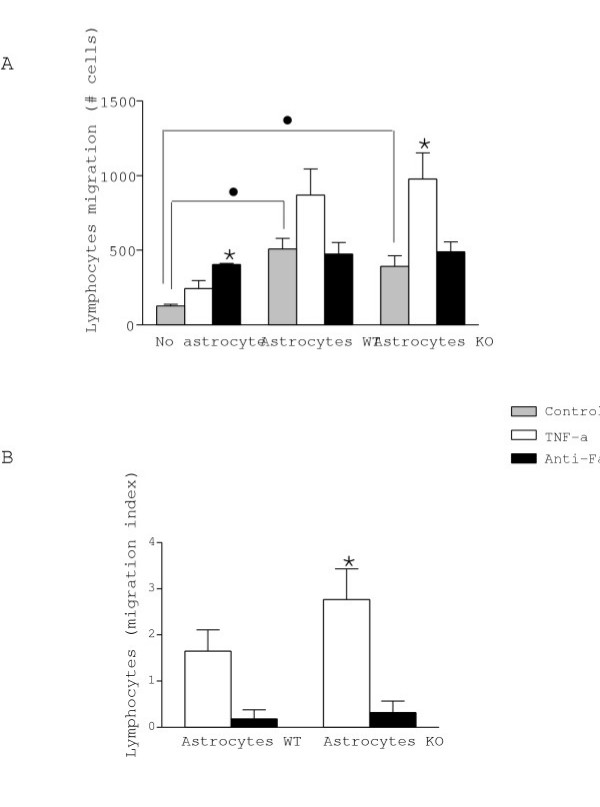
The effect of astrocytes on lymphocyte migration was assessed using Transwell chambers and was expressed by the total number of migrated cells (**A**) and as an index of migration (**B**). Note that anti-Fas treatment alone without astrocytes in the lower compartment induced lymphocyte migration. Only KO astrocytes treated with TNF-α induced significant lymphocyte migration. Values represent the mean +/- s.e.m. of at least three independent experiments. Statistical differences were determined by Student's *t*-test when comparing treatments to control cells (* p < 0.05) or astrocytes vs no astrocyte (● p < 0.05).

MCP-1 is one of the main chemokines involved in the chemoattraction of monocytes and lymphocytes under inflammatory conditions [35,36]. We investigated whether WT astrocytes showing a dramatic upregulation of MCP-1 after exposure to TNF-α or anti-Fas antibody, would differentially affect lymphocyte migration as compared with KO astrocytes whose levels of MCP-1 were unaltered by anti-Fas antibody treatment. The data (Fig. 6) show that astrocytes alone increased by 300% the number of migrating lymphocytes regardless of the genotype. Moreover, in the absence of astrocytes, anti-Fas antibody alone was sufficient to significantly increase (200%) lymphocyte migration. However, the incubation of astrocytes with TNF-α increased the chemotaxis of lymphocytes, in particular in the KO astrocytes. In contrast, no effect of anti-Fas antibody was observed in WT or KO astrocytes as compared to their controls.

## Discussion

The present report shows that TIMP-1 null mutation attenuates the inflammatory response of astrocytes to Fas activation. We show that TIMP-1 null mutation is associated with a higher constitutive MMP-2 expression, and that Fas activation fails to induce MMP-9, ICAM-1 and MCP-1 expression in KO astrocytes as it is the case in WT astrocytes. Moreover, KO astrocytes proliferate less than WT in response to anti-Fas and mrTIMP-1, whereas recruitment of lymphocytes is significantly induced only by TNF-α-treated astrocytes from KO mice.

The constitutive expression of gelatinases in general, and MMP-2 in particular, is higher in KO than in WT astrocytes, suggesting that in the absence of TIMP-1, the baseline regulation of one of its main targets is altered. The overall increase in gelatinase expression along with the absence of TIMP-1-mediated inhibition of MMPs in mutant astrocytes presumably increases MMP activity. As demonstrated previously in other cell types [37] and in brains from TIMP-1 KO mice [38], we assume that TIMP-1 deficiency is not compensated by the overexpression of other TIMPs, at least at the mRNA level. Our data showing a weak decrease of the mitochondrial activity after TNF-α exposure could be interpreted as a mild cytotoxic effect in agreement with studies reporting <20% astrocyte cell death after exposure to the cytokine [39]. We can not rule out the possibility that the absence of cell death under TNF treatment in KO astrocytes may be actually related to a relatively higher baseline proliferative potential in these cultures. We also show that astrocytes are resistant to Fas-mediated cell death in keeping with previous findings in human [21,22] and mouse astrocyte cultures [23]. Moreover, the TNF-α mediated induction of MMP-9 found in mouse astrocytes was equivalent to that reported in rat astrocytes [40]. However, this is the first report demonstrating that the activation of Fas stimulates MMP-9 release by astrocytes. Together, these data support the idea that the activation of receptors of the TNF superfamily triggers inflammatory rather than death signals in astrocytes, and that MMP-9 could act as an inflammatory factor downstream of Fas activation. It is likely that the induced expression of MMP-9 amplifies the inflammatory cascade initiated by cytokines through mechanisms involving the proteolytic activation of latent forms of cytokines. MMP-9 can proteolytically convert latent forms of TNF-α and IL-1β into active forms [24,41]. The presumably pro-inflammatory role of MMP-9 in astrocytes does not preclude its cytotoxic action demonstrated for cortical [42] and hippocampal [43] neurons in excitotoxic/inflammatory paradigms.

The most striking finding of the present study was the reduced response displayed by TIMP-1 deficient astrocytes to anti-Fas antibody, in clear contrast with the response of their WT counterparts. Failure in inducing MMP-9, ICAM-1 and MCP-1 strongly suggests that the absence of TIMP-1 disturbs the Fas signal transduction pathway. TIMPs are considered as multifunctional proteins with anti-MMP, cell-death/survival, and growth promoting activities [44]. Some of the mechanisms underlying these actions are related with the ability of TIMPs to prevent proteolytic processing of membrane receptors, such as death receptors of the TNF superfamily. It has been suggested that the stabilisation of Fas by endogenous (TIMP-1 and TIMP-3) and synthetic MMP inhibitors is responsible for increased sensitivity to apoptosis of various cell types, including Dev neural precursors cells [45] or cancer cells [46]. Recently, MMP-7 has been identified as a sheddase of Fas that induces apoptosis resistance in tumour cells [47]. Considering that astrocytes express high constitutive levels of Fas [23] and that astrocytes deficient for TIMP-1 likely have an increased proteolytic activity, we hypothesise that constitutive cleavage of Fas may indeed reduce the levels of Fas at the membrane, and consequently hamper astrocyte reactivity to anti-Fas antibody treatment. MMP-2, whose levels are constitutively increased in TIMP-1 KO astrocytes, decreased however after anti-Fas treatment. It is tempting to speculate that elevated levels of MMP-2 contribute directly or indirectly to Fas cleavage in normal conditions. The exposure of cells to a Fas-L-like factor would favour the downregulation of MMP-2 in an attempt to re-establish the levels of the receptor. Nevertheless, the role of MMP-2 as a Fas sheddase has not been demonstrated yet.

Together, these data bring support to the hypothesis that TIMP-1 exerts a pro-inflammatory effect by facilitating astrocyte reactivity through the regulation of the MMP/TIMP balance. Astrocyte proliferation is a hallmark of reactivity in inflammatory settings. For the first time, we demonstrate that TIMP-1 induces astrocyte proliferation and this effect is stronger than the effects mediated by TNF-α or anti-Fas antibody. Our data support the idea that TIMP-1 exerts a control on cell growth, as previously suggested by other authors in different cell types [48,4]. Moreover, it has been shown that TIMP-1 accumulates in the nucleus of some cell types [49] and it has been suggested that it could regulate the cell cycle via a MMP-independent mechanism. This idea is reinforced by the finding in our laboratory that mrTIMP-1 increases by 2-fold the number of astrocytes in a mixed neuronal-astrocyte culture, and that this effect is not mimicked by broad spectrum MMP inhibitors (unpublished results). The baseline level of ^3^H-thymidin uptake in KO astrocytes was higher than in the WT. Perhaps, for this reason, a pro-inflammatory stimulus with cytokines or TIMP-1 did not induce further significant increases in KO astrocyte proliferation. It is possible that a low constitutive expression of TIMP-1 maintains astrocytes in a resting "low proliferative" state that reacts promptly to a pro-inflammatory stimulus. On the contrary, the chronic absence of TIMP-1 may cause a state of permanent mild "reactivity" or "inflammatory status" that increases the response threshold for new exogenous pro-inflammatory stimuli. From the above, it follows that TIMP-1 might actually act as a homeostatic factor for astrocytes in the resting state, and as a pro-inflammatory factor in stressful conditions, notably in seizing and ischemic brains, where early and massive induction of TIMP-1 in neurons [5,7] precede astrocyte reactivity. Pro-inflammatory effects of TIMP-1 have also been suggested in *in vivo *models of rheumatoid arthritis [50].

We expected that mutant astrocytes resistant to Fas-mediated induction of MCP-1 would attract lymphocytes less efficiently than WT astrocytes displaying a strong upregulation of MCP-1 in the same treatment conditions. The fact that untreated astrocytes did increase by 300% the number of migrating lymphocytes clearly indicates that these glial cells have a strong chemoattractant capacity in culture conditions, perhaps in part because of their constitutive MCP-1 release. Moreover, the finding that anti-Fas antibody alone caused a 200% increase in lymphocyte migration reveals also a potent chemotactic effect of FasL, independently of its cellular source, in clear contrast with the absence of chemotactic effects of TNF-α alone. These data are coherent with previous reports demonstrating that soluble FasL is chemotactic for neutrophils and that an anti-Fas antibody mimics this effect [51]. In addition, it has been shown that Fas activation stimulates motility in the absence of cytotoxicity [52]. In our experimental model, it its possible that the threshold of stimulation necessary for Fas/astrocyte-mediated lymphocyte migration is already achieved by the individual components of the tandem.

The strong chemoattractant effect of TIMP-1 deficient astrocytes when exposed to TNF-α, cannot be interpreted on the basis of the MCP-1 release, since the latter was equivalent in both WT and TIMP-1 deficient cells. This finding suggests that MCP-1 alone is not sufficient to critically influence the chemoattraction of lymphocytes. The activity of MCPs is regulated by MMP-mediated proteolytic processing, resulting in new bioactive truncated proteins with antagonist effects on leukocyte migration. In this scenario, MMPs would indirectly act as anti-inflammatory agents [34]. However, we cannot speculate on a possible interaction between gelatinases (MMP-2 and MMP-9) and MCP-1 since the latter has been reported to be specifically cleaved by MMP-1 and MMP-3 [34]. An alternative way of interpreting the increased lymphocyte migration in the TIMP-1 deficient astrocytes exposed to TNF-α, refers to the possible regulation of ICAM-1 by gelatinases at the surface of astrocytes; MMPs cleave membrane-bound ICAM-1 in astrocytes [53], and MMP-9 appears to be the key enzyme processing ICAM-1 in tumour cells [54]. Soluble ICAM-1 (sICAM-1) generated by this cleavage has been reported to induce macrophage inflammatory protein-2 (MIP-2) in mouse astrocytes and subsequently stimulate leucocytes migration [55]. A lower TIMP-1 inhibitory capacity in the mutant astrocytes, along with an increased gelatinase activity, would favour sICAM-1 proteolytic generation and possibly lymphocyte migration after TNF-α treatment. This idea is coherent with the overall lower ICAM-1 levels found in the TIMP-1 KO mice.

## Conclusion

In summary, the present data provide evidence linking the MMP/TIMP balance to the reactive response of astrocytes following pro-inflammatory stimuli. The control of astrocyte reactivity by the MMP/TIMP axis affects in a particular way the Fas/FasL pathway, and suggests that in astrocytes, the outcome of the neuroinflammatory status in pathological situations may involve the mutual regulation of these two molecular systems. We can not exclude that other MMP substrates (ICAM, MCPs) are also affected by changes in the proteolytic balance. We are just starting to unveil the importance that the proteolytic modification of bioactive factors may have in the control of cell behaviour and intercellular communication. The study of the MMP/TIMP roles in neural cells and of the mechanisms whereby these proteinases and inhibitors exert the control of cell behaviour is a challenging question that needs further investigation.

## Methods

### Animals

All experimental procedures were performed in compliance with our institutional guidelines after obtaining the authorisation of the Laboratory Animal Committee of the Medical Faculty.

TIMP-1 deficient mice (Sv-129) were generated by homologous recombination and functional TIMP-1 protein was detected in WT cells, but not in co-isogenic cell lines carrying the mutation in *Timp1 *[13]. Genetically modified (KO) and WT mice have an identical genetic background. Genomic DNA was extracted from the tail tips of mice to determine their genotype by PCR.

### Astrocyte cultures

Astrocytes were obtained from the brains of 2-day-old mice. After removal of the meninges, the brains were dissociated into a single-cell suspension by trypsinisation and mechanical disruption. The cells were seeded on poly-L-lysine (Sigma St Louis, MO, USA) coated culture plates at the density of 4 × 10^4 ^cells per cm^2 ^and grown at 37°C in a 5% CO_2 _humidified atmosphere in Dulbecco's Modified Eagle Medium (DMEM) containing 10% foetal calf serum, 2 mM L-glutamine, penicillin (100 U/ml) and streptomycin (100 μg/ml) (all from InVitrogen, Carlsbad, CA, USA). The medium was replaced every 3 days. After 8–10 days *in vitro*, the medium was changed for 24 h into serum free medium containing DMEM, 2 mM L-glutamine, penicillin (100 U/ml), streptomycin (100 μg/ml), 5 μg/ml insulin (Sigma,) and 0,05% bovine serum albumin (BSA – InVitrogen). Cells were then treated for 24 h with 10 ng/ml TNF-α (R&D Systems, Minneapolis, MN, USA) and 0.5 μg/ml hamster anti-mouse Fas antibody (Clone Jo2, BD Pharmingen, San Jose, CA, USA) which mimics the effects of FasL, activates Fas and stimulates pro-inflammatory responses from astrocytes [23].

### Western blot

Protein concentration of culture supernatants was normalised using the Lowry method (DC-Protein assay, Bio-Rad, Hercules, CA, USA). Using a Bio-Rad MiniBlot system, equal amounts of protein were subjected to 8.5% sodium dodecyl sulphate – polyacrylamide gels electrophoresis (SDS-PAGE), under reducing and denaturing conditions, and then transferred to PVDF immunoblotting membrane (Amersham Pharmacia Biotech, Buckinghamshire, UK). TIMP-1 was detected by a goat anti-mouse TIMP-1 antibody (R&D Systems), followed by biotinylated anti-goat IgG and streptavidin-peroxidase (both from Jackson ImmunoResearch, West Grove, PA, USA), and then visualised by chemiluminescence reaction using the ECL system (Amersham). Glycosylated mouse recombinant (mr) TIMP-1 (R&D Systems) was used as a control in each gel.

### Astrocyte and microglia staining

In order to charaterise the cell type composition of our cultures we used glial fibrillary acidic protein (GFAP) as an astrocytic marker and F4/80 as a microglial marker. Briefly, cells were preincubated with 0.1% triton X-100 and 5% normal goat serum (NGS) for 30 min, incubated with rabbit anti-cow GFAP (DAKO, Glostrup, Denmark) (1/500) and rat anti-mouse F4/80 (Serotec, Kidlington, Oxford, UK) (1/500) antibodies diluted in PBS containing 0.1% triton X-100 and 5% serum for 1 h. Cells were then rinsed in PBS, incubated for 1 h with Alexa Fluor^® ^594 anti-rabbit and Alexa Fluor^® ^488 anti-rat antibodies respectively (Molecular Probes, Eugene, OR, USA), rinsed in PBS, and the DNA intercalant Hoechts #33258 (Molecular Probes) was added during 5 min to label cell nuclei. Cells were then mounted in fluorescent mounting medium (DAKO), observed under a Nikon E800 upright microscope (Nikon Champigny-sur-Marne, France) equipped with epifluorescence, TRITC, FITC and DAPI filters, DXM 1200 camera and the LUCIA image analysis software.

### 3-(4,5-Dimethylthiazol-2yl)-2,5-diphenyl tetrazolium bromide (MTT) assay

In order to test a possible cytotoxic effect of treatments, TNF-α and anti-Fas antibody were incubated for 24 h and cell death measured by the MTT (Sigma) assay that detects mitochondrial activity in living cells [56]. Results are expressed as the arbitrary units of optical density. The means ± S.E.M. were obtained from triplicates within a single experiment and at least three independent experiments.

### Gel zymography

Equal amounts of protein were subjected to 8.5% SDS-PAGE containing 0.5% gelatine (Sigma) in non-denaturing, non-reducing conditions. Gels were washed twice for 15 min in 2.5% Triton X-100 to remove SDS, and incubated at 37°C for 22 h in 100 mM Tris pH 7.4 and 15 mM CaCl_2_. Gels were then stained with 0.1% Coomassie blue R-250 (Bio-Rad) for 20 min and distained in 10% acetic acid until clear bands of gelatinolysis appeared on a dark background. Bacterial collagenase (10^-2 ^U/ml, Sigma) was loaded in each gel as a control and used for normalisation. Gels were scanned and bands were quantified using GeneTools software. Each test was performed in triplicate within single experiments and means ± S.E.M. were obtained from at least three independent experiments. Some zymogram gels were incubated with 1 mM 1,10-*0*-phenanthroline (Sigma) or BB-3103, two broad spectrum inhibitors of metalloproteinases.

### Flow cytometry

Astrocyte cultures were incubated on ice for 15 min with the rat anti-mouse ICAM-1 antibody purified from the supernatants of YN1 hybridoma cells (R&D Systems). After washes, cells were treated with FITC-labelled goat anti-rat IgG (Jackson ImmunoResearch) for 15 min in ice. Cells were washed, centrifuged and resuspended in PBS and 2% paraformaldehyde. A control without anti-ICAM-1 antibody served as blank to normalise the mean of fluorescence. FACS analysis was performed on a FACScalibur (Becton Dickinson, Franklin Lakes, NJ, USA) using Cellquest Software (Becton Dickinson). The number of positive cells in each assay was standardised to 5000. Results were expressed in arbitrary units corresponding to the mean of fluorescence, and were obtained from at least three independent experiments.

### Enzyme linked immuno-adsorbent assay for MCP-1

MCP-1 expression in astrocyte supernatants was measured using an ELISA kit (BD Pharmingen) according to the manufacturer's instructions. Equal amounts of protein were added onto anti-mouse MCP-1 antibody-coated wells, detected by horseradish peroxydase-conjugated anti-mouse MCP-1 antibody and revealed with o-phenylenediamine. Recombinant mouse MCP-1 was used as control to determine the concentration of MCP-1 in the sample. Each test was performed in triplicate within single experiments and means ± S.E.M. were obtained from at least three independent experiments.

### Proliferation assay by ^3^H-thymidin incorporation

After 8–10 days *in vitro*, astrocytes were trypsinised and reseeded in serum containing medium in 96-well flat-bottom plates, at the density of 10^4 ^cells per well. Twenty four hours after, the culture medium was replaced with serum-free medium for 24 h and cells were then treated with TNF-α, anti-Fas antibody or mrTIMP-1 in triplicate. Two days later, 1 μCi of ^3^H thymidin (Amersham Pharmacia Biotech) was added in each well. After 18 h, cells were harvested on filter mats, dried and counted using 1600TR liquid scintillation analyser (Pachard Instruments, Meriden, CT, USA). The results were expressed as total cpm. Each test was performed in triplicate within single experiments and means ± S.E.M. were obtained from at least three independent experiments.

### Lymphocyte recruitment in transwell chambers

We used a transwell system in order to evaluate the influence of astrocytes treated with TNF-α and anti-Fas antibody on lymphocyte migration. Lymphocytes were obtained from spleen, cultured at 4 × 10^4 ^cells per cm^2 ^and activated with recombinant IL-2 (InVitrogen) and with the supernatant of splenic cells stimulated with concanavalin A (Sigma). Astrocytes were seeded in the lower part of the well, as described above, and treated for 24 h with TNF-α or anti-Fas antibody in a serum free medium. Lymphocytes were labelled with 10 μM CFSE (5-(and-6)-carboxyfluorescein diacetate, succinimidyl ester (5(6)-CFDA, SE; CFSE – Molecular Probes) by a 15 min incubation. Lymphocytes (1.5 × 10^4 ^per well) were then seeded on the upper part of 3 μm pore clear polycarbonate transwells (Costar, Cambridge, MA, USA) and allowed to migrate for 7 hours. The filters were then removed and photomicrographs of migrated lymphocytes in the lower well were taken using a Nikon TE300 inverted microscope equipped a FITC filter, and images were analysed using a DXM 1200 camera and the LUCIA image analysis software (Nikon). Wells without astrocytes in the lower part or astrocytes without treatment were used as controls. The number of labelled lymphocytes in the lower compartment was quantified using GenTools software (Syngene). Tests were performed in triplicate within single experiments and means ± S.E.M. were obtained from three independent experiments.

## List of abbreviations

CNS, central nervous system; ICAM-1, intercellular adhesion molecule-1; MCP-1, monocyte chemoattractant protein-1; MMP, matrix metalloproteinase; TIMP, tissue inhibitor of MMP; TNF, Tumour Necrosis Factor.

## Authors' contributions

CO performed most of the assays and co-wrote the manuscript.

RC participated to the lymphocyte recruitment assay.

JB made substantial contributions to conception and design of experiments.

PS provided the transgenic mice.

MK contributed to design the project and revised the manuscript for important intellectual content.

SR supervised the project and wrote the manuscript.

All authors read and approved the final manuscript and its submission to BMC Neuroscience.
